# Exposure to an Extremely-Low-Frequency Magnetic Field Stimulates Adrenal Steroidogenesis via Inhibition of Phosphodiesterase Activity in a Mouse Adrenal Cell Line

**DOI:** 10.1371/journal.pone.0154167

**Published:** 2016-04-21

**Authors:** Kazuyoshi Kitaoka, Shiyori Kawata, Tomohiro Yoshida, Fumiya Kadoriku, Mitsuo Kitamura

**Affiliations:** 1 Institute of Liberal Arts and Sciences, Tokushima University, Tokushima, Japan; 2 Student Lab, Tokushima University Faculty of Medicine, Tokushima, Japan; 3 Support Center for Advanced Medical Sciences, Institute of Biomedical Sciences, Tokushima University Graduate School, Tokushima, Japan; North Carolina State University, UNITED STATES

## Abstract

Extremely low-frequency magnetic fields (ELF-MFs) are generated by power lines and household electrical devices. In the last several decades, some evidence has shown an association between ELF-MF exposure and depression and/or anxiety in epidemiological and animal studies. The mechanism underlying ELF-MF-induced depression is considered to involve adrenal steroidogenesis, which is triggered by ELF-MF exposure. However, how ELF-MFs stimulate adrenal steroidogenesis is controversial. In the current study, we investigated the effect of ELF-MF exposure on the mouse adrenal cortex-derived Y-1 cell line and the human adrenal cortex-derived H295R cell line to clarify whether the ELF-MF stimulates adrenal steroidogenesis directly. ELF-MF exposure was found to significantly stimulate adrenal steroidogenesis (p < 0.01–0.05) and the expression of adrenal steroid synthetic enzymes (p < 0.05) in Y-1 cells, but the effect was weak in H295R cells. Y-1 cells exposed to an ELF-MF showed significant decreases in phosphodiesterase activity (p < 0.05) and intracellular Ca^2+^ concentration (p < 0.01) and significant increases in intracellular cyclic adenosine monophosphate (cAMP) concentration (p < 0.001–0.05) and cAMP response element-binding protein phosphorylation (p < 0.05). The increase in cAMP was not inhibited by treatment with NF449, an inhibitor of the Gs alpha subunit of G protein. Our results suggest that ELF-MF exposure stimulates adrenal steroidogenesis via an increase in intracellular cAMP caused by the inhibition of phosphodiesterase activity in Y-1 cells. The same mechanism may trigger the increase in adrenal steroid secretion in mice observed in our previous study.

## Introduction

Exposure to extremely low-frequency magnetic fields (ELF-MFs), which are mainly generated by power lines and household electrical devices, has increased in modern society. Multiple studies using cell lines, animal models, and human subjects and also epidemiological studies have investigated whether ELF-MFs have biological effects; however, the effects remain controversial [1–3]. In the last several decades, an association between ELF-MF exposure and depression and/or anxiety has been reported in epidemiological [4, 5] and animal studies [6, 7]. Early animal studies also reported a relationship between ELF-MF-induced depressive behavior and adrenal steroid secretion, and the mechanism of adrenal steroidogenesis is assumed to involve induction of a common stress response resulting from ELF-MF exposure [7, 8].

We previously reported that chronic ELF-MF exposure affects corticosterone synthesis and depression-like behavior without enhancement of the hypothalamic–pituitary–adrenal axis in mice [9]. This result suggests that ELF-MF stimulates steroidogenesis in the adrenal cortex directly, and not via the common stress response. However, the mechanism underlying ELF-MF-triggered steroidogenesis is not known at present.

In this study, we first investigated the effect of ELF-MF exposure on the mouse adrenal cortex-derived Y-1 cell line to clarify whether ELF-MF stimulates adrenal steroidogenesis directly. We found that adrenal steroidogenesis was enhanced in Y-1 cells by ELF-MF exposure. Additionally, we investigated the mechanism underlying the steroidogenesis. The possibility of an effect on humans was estimated using the adrenal cortex-derived H295R cell line; no effects were observed in these cells. Our results showed that the ELF-MF directly stimulated steroidogenesis in the adrenal cortex and not via the common stress response in the mouse adrenal cell line.

## Materials and Methods

### Cell culture

The mouse adrenal cortex-derived Y-1 cell line (RBRC-RCB533; provided by RIKEN BRC through the National Bio-Resource Project of the MEXT, Tsukuba, Japan) and the human adrenal cortex-derived H295R cell line (ATCC CRL-2128; provided by American Type Culture Collection, Manassas, VA, USA) were used. The cells were cultured in 35-mm diameter culture dishes. Y-1 cells were seeded in RPMI 1640 medium + 10% fetal bovine serum at a density of 2 × 10^5^ cells/dish and incubated at 37 ± 0.5°C in a 5% CO_2_ humidified atmosphere for 4 days. H295R cells were seeded in DMEM:F12 medium + 2.5% Nu-serum I and 1% ITS+mix (BD Biosciences, Bedford, MA, USA) at density of 8 × 10^5^ cells/dish and incubated at 37 ± 0.5°C in a 5% CO_2_ humidified atmosphere for 4 days.

### ELF-MF exposure

Confluency was achieved after the 4-day incubation. Then, the cell dishes in the ELF-MF group were exposed to a 60-Hz ELF-MF for 1–48 h by using a custom-made exposure apparatus after medium replacement. In the sham control group, sham exposure was performed simultaneously with the ELF-MF exposure using a sham apparatus, which was made using the same materials with the same size, except for the coil. Details regarding the ELF-MF and sham exposure apparatuses have been provided in a previous study [10]. In brief, each exposure apparatus consisted of a cylindrical plastic (polyvinyl chloride) container (37 cm outer diameter; 34.5 cm inner diameter; 33 cm length) placed vertically. In the ELF-MF exposure apparatus, the coil consisted of one primary coil and two secondary coils that were placed on the upper and lower ends of the primary coil. The coils were made of enameled wires (2.12 mm diameter, electric resistance 5.66 Ω/km). For the primary coil, the wire was wound 150 times around the plastic container. For the two secondary coils, the wire was wound 33 times around every layer to correct the uneven distribution of the magnetic flux density of the primary coil. A plastic incubator (25-cm length, 20-cm width, and 10-cm height) containing the culture dishes was placed in the middle of each plastic container. To maintain a temperature of 37 ± 0.5°C in a 5% CO_2_ atmosphere in the incubator, warm water (40 ± 0.5°C) and 5% CO_2_ air were supplied in the container. The temperature difference between ELF-MF and sham exposure apparatuses was maintained within ± 0.5°C by the flow of warm water in each apparatus. To maintain humidity, distilled water was arranged in the incubator. The base values of the ELF-MF and static MF in the ELF-MF and sham exposure apparatuses were 40–50 μT and 25–30 μT, respectively. The intensity of the ELF-MF was set between 0.25 and 3 mT. The intensity of MF was measured using a gaussmeter (type 501; Nihon Denji Sokki, Tokyo, Japan). After exposure, the medium and cells were sampled and used for subsequent analysis.

### Adrenal steroid quantification

Adrenal steroids (corticosterone, aldosterone, and cortisol) secreted in the medium were quantified using ELISA kits (500655, 10004377, 500360, respectively; Cayman Chemical; Ann Arbor, MI, USA). The value was standardized (/10^5^ cells) according to the cell count, which was estimated by C-Chip (DHC-B02; NanoEnTek, Seoul, Korea), in each sampled dish. The experiments were performed according to the manufacturer’s instructions. Steroids were quantified twice per sample and the data were averaged.

### Real-time reverse transcription polymerase chain reaction (qRT-PCR)

mRNA extraction and subsequent two-step qRT-PCR analysis were performed as reported previously [9]. Briefly, total RNA was extracted from the cells of each dish with ISOGEN reagent (Nippon Gene, Toyama, Japan). Reverse transcription of total RNA was performed using a Transcriptor First Strand cDNA Synthesis Kit (Roche Diagnostics, Mannheim, Germany). The cDNA was quantified using Universal ProbeLibrary probes (Roche Diagnostics) and a Prism 7500 apparatus (Applied Biosystems, Foster, CA). The PCR program was as follows: 10 min pre-heating at 95°C and 55 cycles at 94°C for 30 s, 57°C for 30 s, and 72°C for 45 s. Expression of glyceraldehyde-3-phosphate dehydrogenase (*Gapdh*) was used as an endogenous control for quantification of the mRNA targets (threshold cycle (Ct) of target expression/Ct of *Gapdh* expression). The target genes, Universal ProbeLibrary probe numbers, and primer sequences used in this study are shown in Table 1. Quantification was performed once for each sample and gene target.

**Table 1:** Genes quantified in this study, along with the corresponding primer sequences and probe numbers from the Universal ProbeLibrary.

Gene	NCBI RefSeq number	Sense base numbers	Sense sequence	Universal ProbeLibrary	Antisense base numbers	Antisense sequence
Mouse steroidogenic acute regulatory protein (*Star*)	NM_011485.4	765–786	5'-aaactcacttggctgctcagta-3'	#83	819–838	5'-tgcgataggacctggttgat-3'
Mouse cytochrome P450, family 11, subfamily a, polypeptide 1 (*Cyp11a1*)	NM_019779.3	1381–1398	5'-gggtgttcggcagtgtct-3'	#78	1468–1489	5'-cacatcacggagattttgaact-3'
Mouse cytochrome P450, family 11, subfamily b, polypeptide 1 (*Cyp11b1*)	NM_001033229.2	708–729	5'-aaaccaccacacagctcttgta-3'	#92	812–836	5'-acacattcttgatacatttggtgac-3'
Mouse cytochrome P450, family 11, subfamily b, polypeptide 2 (*Cyp11b2*)	NM_009991.3	267–285	5'-gcagggccaagaaaaccta-3'	#80	329–348	5'-tttccctacactgtgcctga-3'
Mouse glyceraldehyde-3-phosphate dehydrogenase (*Gapdh*)	NM_008084.2	763–781	5'-tgtccgtcgtggatctgac-3'	#80	818–837	5'-cctgcttcaccaccttcttg-3'
Human steroidogenic acute regulatory protein (*Star*)	NM_000349.2	307–325	5'-agacacatgcgcaacatga-3'	#17	396–415	5'-ggttaatccacgtgctaggg-3'
Human cytochrome P450, family 11, subfamily a, polypeptide 1 (*Cyp11a1*)	NM_000781.2	559–580	5'-gacccataggagtcctgttgaa-3'	#79	635–652	5'-ttcttggtggcctctgga-3'
Human cytochrome P450, family 11, subfamily b, polypeptide 1 (*Cyp11b1*)	NM_000497.3	519–538	5'-ccctgaagaagaaggtgctg-3'	#26	606–625	5'-tccaaaaagagccaagttgc-3'
Human cytochrome P450, family 11, subfamily b, polypeptide 2 (*Cyp11b2*)	NM_000498.3	200–218	5'-tggagatgcaccagacctt-3'	#17	242–261	5'-tggtcctcccaagttgtacc-3'
Human cytochrome P450, family 17, subfamily a, polypeptide 1 (*Cyp17a1*)	NM_000102.3	566–584	5'-tttgccctgttcaaggatg-3'	#78	627–646	5'-ggccagcatatcacacaatg-3'
Human glyceraldehyde-3-phosphate dehydrogenase (*Gapdh*)	NM_002046.3	83–101	5'-agccacatcgctcagacac-3'	#60	130–148	5'-gcccaatacgaccaaatcc-3'

### Western blotting

Protein extraction and subsequent protein expression analysis were performed as described previously [11]. The cells were lysed using RIPA buffer mixed with protease inhibitor and phosphatase inhibitor (EzRIPA Lysis kit; Atto, Tokyo, Japan). Supernatant-derived protein samples (10 mg of protein per sample) were electrophoresed on a 2–20% sodium dodecyl sulphate polyacrylamide gel and transferred to a polyvinylidene difluoride membrane (Clearblot P membrane; Atto, Tokyo, Japan). The membrane was blocked in blocking reagent (EzBlock; Atto) at room temperature for 30 min and incubated sequentially (4°C, overnight, each incubation) with mouse polyclonal antibodies (1:5000 dilution) against StAR (#8449; Cell Signaling Technology, Danvers, MA, USA), CYP11A1 (#12491; Cell Signaling Technology), CYP11B2 (SAB1303149; Sigma-Aldrich, St. Louis, MO, USA), CYP17A1, total cAMP response element-binding protein (CREB) (#9197, Cell Signaling Technology), phospho-CREB (#9198, Cell Signaling Technology), and actin (A2066; Sigma-Aldrich), followed by a 1-h incubation with a horseradish-peroxidase-conjugated sheep antibody against rabbit IgG (A602PN, 1:20,000 dilution; American Qualex, San Clemente, CA, USA). The CYP17A1 antibody was kindly provided by Dr. Kazunori Ishimura (Professor Emeritus, Tokushima University). Immunoreactive protein bands were developed using a chemiluminescent HRP substrate (Immobilon Western; Millipore, Billerica, MA, USA). The bands were scanned and analyzed using a computer-based image analyzer (LAS-3000UVmini; Fujifilm, Tokyo, Japan). The intensity of the bands corresponding to each sample was normalized to that of the band corresponding to actin for enzyme expression. For analysis of CREB phosphorylation, the intensity was normalized to total CREB expression. Each sample and protein target was analyzed once.

### Cyclic adenosine monophosphate (cAMP) quantification

Intracellular cAMP was quantified using an ELISA kit (581001; Cayman Chemical). The value was standardized to the amount of total protein in each extract, which was estimated using the Pierce BCA Protein Assay Kit (Thermo Fisher Scientific, Waltham, MA, USA). The experiments were performed according to the manufacturer’s instructions. cAMP was quantified twice per sample, and the data were averaged.

### NF449 treatment

NF449 (4, 4', 4'', 4‴- [carbonylbis[imino- 5, 1, 3- benzenetriylbis(carbonylimino)]]tetrakis- 1, 3- benzenedisulfonic acid; Cayman Chemical), an inhibitor of the Gsα subunit of G protein, was dissolved in culture medium (10 or 30 μM). Normal culture medium was used for the vehicle control. NF449 or vehicle control was added to the each culture dish before 6-h ELF-MF or sham exposure. After exposure, the intracellular cAMP concentration was estimated.

### Fura-2AM assay

The change in intracellular Ca^2+^ concentration ([Ca^2+^]_i_) in Y-1 cells was estimated using the Fura-2AM kit (Calcium Kit-Fura2; Dojindo Molecular Technologies, Kumamoto, Japan). Y-1 cells were seeded in RPMI 1640 medium + 10% fetal bovine serum at a density of 2 × 10^4^ cells/well in 96-well culture plates and incubated at 37 ± 0.5°C in a 5% CO_2_ humidified atmosphere for 4 days. After pre-incubation and medium replacement, 24-h ELF-MF and sham exposure were performed. Next, 5 μM Fura2-AM was added to each well for 1 h at 37 ± 0.5°C according to the manufacturer’s instructions. Fluorescence analysis was performed with excitation wavelengths of 340 nm and 380 nm and an emission wavelength of 510 nm with a microplate reader (Varioskan Flash; Thermo Fisher Scientific) in each well. The 340/380 nm emission ratio was used for comparative assessment of [Ca^2+^]_i_. The Fura ratio was estimated once for each sample.

### Phosphodiesterase (PDE) activity

Cells were lysed using RIPA buffer mixed with a protease inhibitor and a phosphatase inhibitor. The supernatant was purified using chromatography columns (Micro Bio-Spin P-6; Bio-Rad, Hercules, CA, USA). The PDE activity of the desalted samples was estimated using the PDE activity assay kit (BML-AK800; Enzo Life Sciences, Farmingdale, NY, USA). The value (amount of 5' AMP) was standardized to the amount of total protein, which was estimated using the Pierce BCA Protein Assay Kit (Thermo Fisher Scientific) in each sample. PDE quantification was conducted twice per sample, and data were averaged.

### Statistical analysis

Data have been provided as the mean ± standard error of the mean (S.E.M.). Repeated measures two-way analysis of variance was used to analyze the non-standardized sequential data (steroids and cAMP concentration, and PDE activity). When ANOVA showed significance in groups, post hoc Scheffe’s test was performed. An unpaired t-test was used to compare standardized sham and ELF-MF data (gene and protein expression, Fura2-AM assay, and ratios relative to sham exposure for the intensity-dependent effect of ELF-MF exposure). Significance was set at p < 0.05.

## Results

### Effects of ELF-MF exposure on adrenal steroid secretion and adrenal enzyme expression

**Fig 1:**
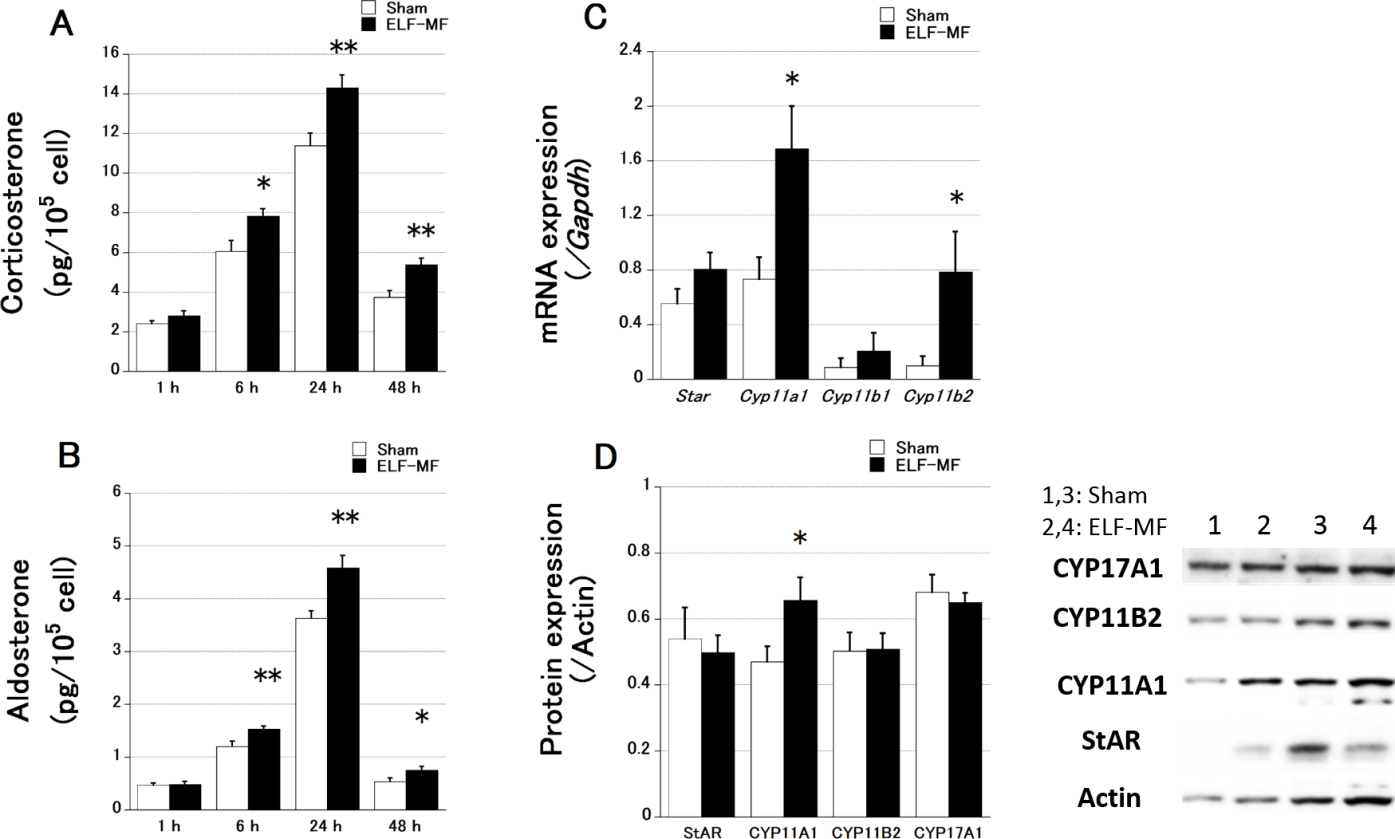
Effects of ELF-MF and sham exposure on steroid secretion and enzyme expression in Y-1 cells. Data are presented as the mean ± S.E.M. Corticosterone (A) and aldosterone (B) levels were significantly higher after 6 h of ELF-MF exposure. (C) *Cyp11a1* and *Cyp11b2* mRNA levels showed a significant increase after 24 h of exposure to the ELF-MF as indicated by qRT-PCR results. (D) Secreted CYP11A1 protein levels were significantly increased as indicated by western blotting. A representative blot image is shown (lanes 1 and 3: sham-exposed samples; lanes 2 and 4: ELF-MF- exposed samples). Steroids were quantified twice per sample (culture dish), and qRT-PCR and western blotting were performed once. n = 8 each, *p < 0.05, **p < 0.01 vs. sham exposure.

Fig 1 shows the effects of different periods of exposure to a 1.5-mT ELF-MF on adrenal steroid secretion and the expression of adrenal enzymes in mouse adrenal Y-1 cells (n = 8 each). Corticosterone and aldosterone secretion increased until 24 h and was then reduced at 48 h of ELF-MF and sham exposure. The change in steroid secretion over time was significant (p < 0.0001). However, the levels after 6, 24, and 48 h of ELF-MF exposure were significantly higher than those after sham treatment (Fig 1A and 1B).

Next, we estimated the mRNA and protein expression of adrenal steroid synthetic enzymes (StAR, CYP11A1, CYP11B1, and CYP11B2) to clarify the cause of the increase in adrenal steroid levels after 24-h ELF-MF and sham exposure. StAR facilitates the movement of cholesterol from the outer to the inner mitochondrial membrane [12]. CYP11A1 is a rate-limiting enzyme for steroidogenesis [13] [14], and CYP11B1 and CYP11B2 convert 11-deoxycorticosterone to corticosterone and aldosterone [15]. *Cyp11a1* and *Cyp11b2* mRNA levels were significantly higher after 24-h exposure to the ELF-MF than after 24-h sham exposure, whereas no differences were noted for *Star* and *Cyp11b1* mRNA (Fig 1C). At the protein level, a significant difference was noted only for CYP11A1 (Fig 1D).

**Fig 2:**
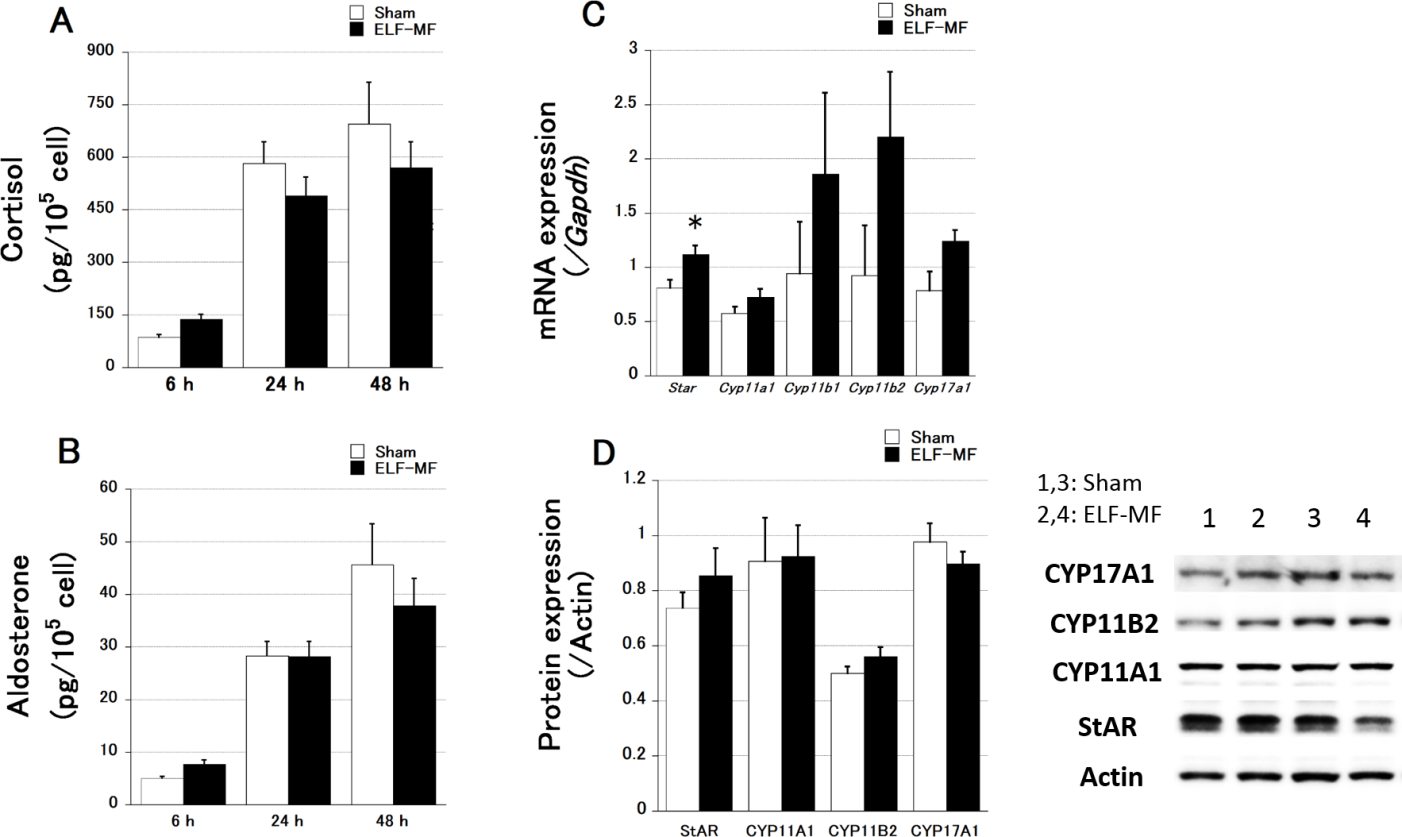
Effects of ELF-MF and sham exposure on steroid secretion and enzyme expression in H295R cells. All data are presented as the mean ± S.E.M. Cortisol (A) and aldosterone (B) secretion did not show a significant difference between sham and ELF-MF exposure. (C) After 24 h of ELF-MF and sham exposure, mRNA expression of *Star* showed a significant increase in ELF-MF-exposed cells. (D) No significant increase was observed in protein levels. A representative blot image is shown (lanes 1 and 3: sham-exposed sample; lanes 2 and 4: ELF-MF-exposed sample). Steroid levels were quantified twice per sample (culture dish), and qRT-PCR and western blotting were performed once. n = 8 each, *p < 0.05, **p < 0.01.

The results for adrenal steroid secretion and adrenal enzyme expression in human adrenal H295R cells (n = 8 each) after different periods of exposure to a 1.5-mT ELF-MF are summarized in Fig 2. The levels of cortisol and aldosterone secretion were not significantly different between sham and ELF-MF exposure (Fig 2A and 2B). The increase in steroid secretion overt time was significant (p < 0.0001). mRNA expression of *Star* was significantly higher under the ELF-MF than under sham exposure (Fig 2C), whereas differences for the other adrenal steroid synthetic enzymes were not significant. No significant differences were found at the protein level (Fig 2D).

### Effects of ELF-MF exposure on cAMP concentration, CREB phosphorylation, and [Ca^2+^]_i_

**Fig 3:**
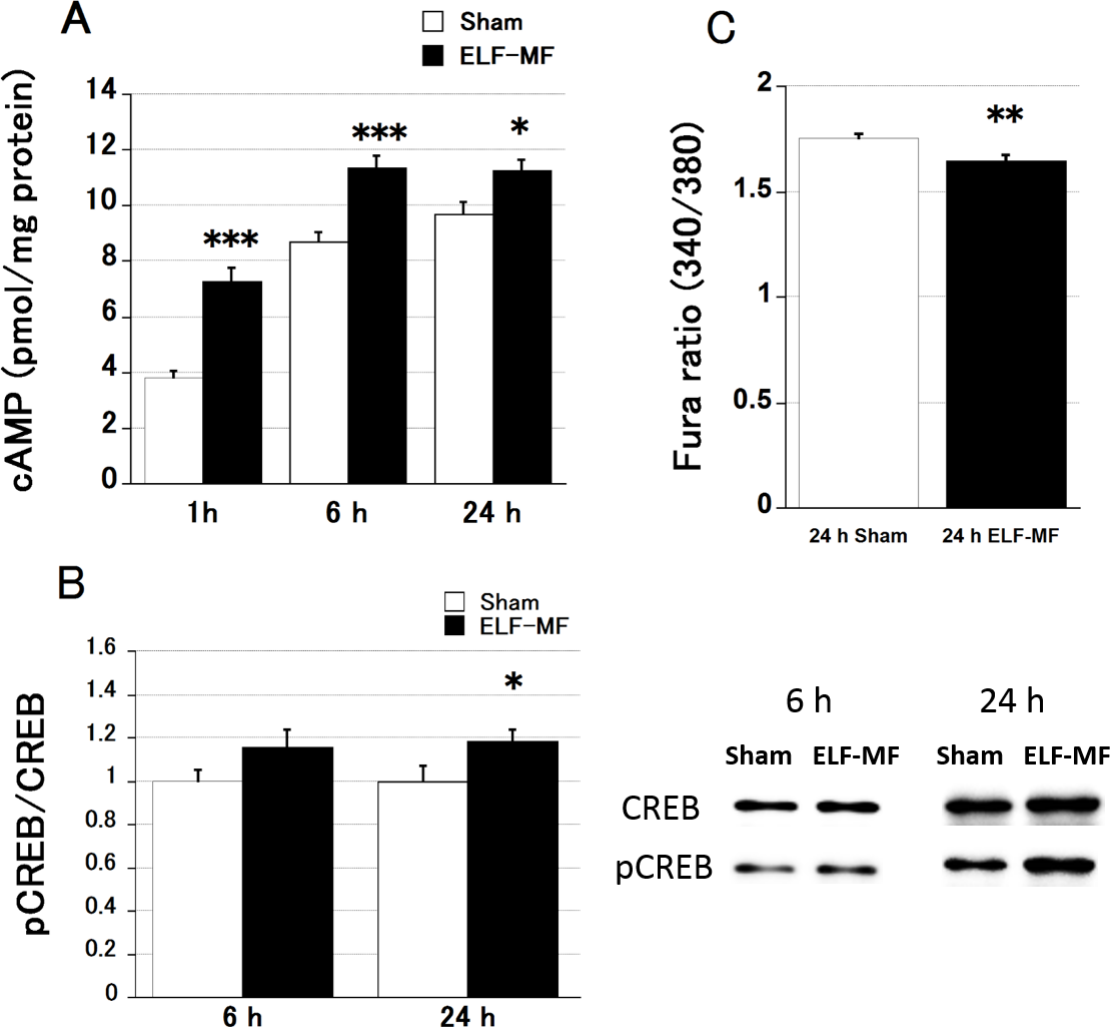
Intracellular cAMP concentration was estimated in Y-1 cells exposed to ELF-MF and sham treatments. All data are presented as the mean ± S.E.M. (A) cAMP levels were significantly higher upon exposure to a 1.5-mT ELF-MF than after sham exposure for all durations (n = 8 each). (B) CREB phosphorylation was significantly higher in the ELF-MF group than in the sham group with 24 h of exposure (n = 8 each). (C) The 340/380 nm emission Fura ratio, which indicates [Ca^2+^]_i_, was significantly lower in cells exposed to 24-h ELF-MF treatment (n = 10) than in sham-exposed cells (n = 10). cAMP levels were quantified twice per sample (dish). The Fura ratio was estimated once per sample. *p < 0.05, **p < 0.01, ***p < 0.001.

Next, we investigated the mechanism underlying the adrenal steroidogenesis induced by ELF-MF exposure in Y-1 cells. Adrenal steroidogenesis is regulated by two signaling pathways: the adrenocorticotropic hormone (ACTH)-stimulated pathway and the angiotensin II-stimulated pathway [16]. To determine which pathway is involved in ELF-MF-induced adrenal steroidogenesis in Y-1 cells, we analyzed intracellular cAMP levels, CREB phosphorylation, and [Ca^2+^]_i_ after different periods of exposure to a 1.5-mT ELF-MF or sham treatment. cAMP levels increased significantly over time (p < 0.0001). Exposure to ELF-MF for 1, 6, and 24 h resulted in significantly higher cAMP levels than sham exposure for these periods (Fig 3A). CREB phosphorylation was significantly higher upon exposure to ELF-MF than after sham exposure for 24 h (Fig 3B). Conversely, intracellular Ca^2+^ levels were significantly lower after 24-h ELF-MF exposure than after sham treatment (Fig 3C). Taken together, these results indicate that the PKA pathway is involved in ELF-MF-induced adrenal steroidogenesis in Y-1 cells.

### Effects of NF449 treatment on cAMP concentration and PDE activity after ELF-MF exposure

**Fig 4:**
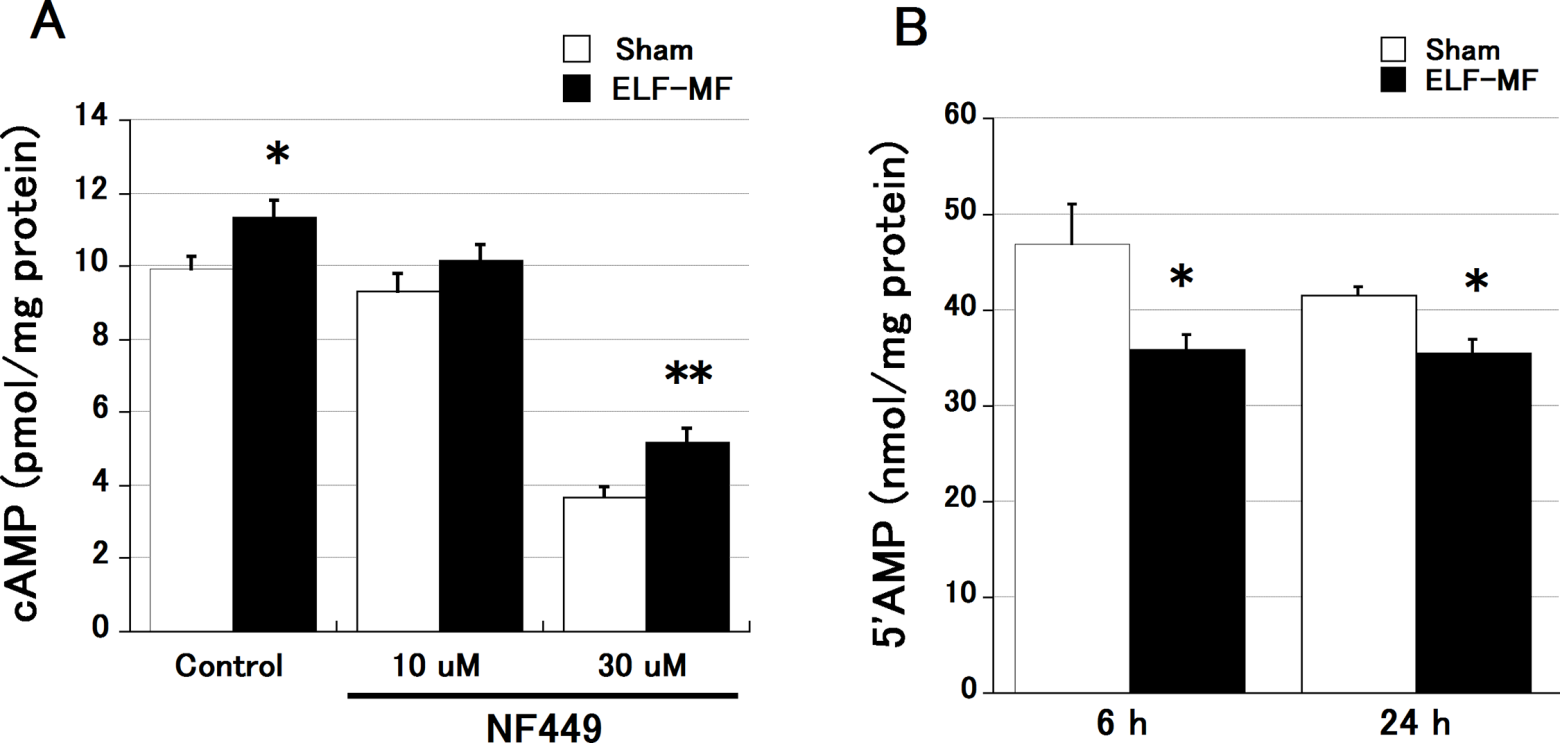
Effect of NF449 on the increase in cAMP concentration resulting from ELF-MF exposure. All data are presented as the mean ± S.E.M. (A) NF449 inhibited the increase in cAMP concentration in Y-1 cells exposed to sham treatment and 1.5 mT ELF-MF for 6 h; however, the cAMP-increasing effect of ELF-MF was still significantly higher than that of the sham treatment after 30 μM NF449 treatment (n = 8 each). (B) PDE activity was significantly lower in ELF-MF- than in sham-exposed Y-1 cells at both 6 h and 24 h (n = 4 each). cAMP and PDE were quantified twice per sample. *p < 0.05, **p < 0.01.

To clarify the mechanism underlying the ELF-MF-induced increase in intracellular cAMP levels, we investigated the effect of NF449, a selective Gsα-subunit G-protein antagonist inhibiting cAMP production, on the increase in cAMP resulting from 1.5-mT ELF-MF exposure and on PDE activity in cells exposed to the ELF-MF for 6 h (Fig 4). NF449 inhibited the increase in the cAMP concentration in Y-1 cells exposed to sham treatment and 1.5 mT ELF-MF for 24 h (p< 0.0001). However, the significant cAMP increase resulting from ELF-MF seen in the vehicle control was preserved after treatment with 30 μM NF449 (Fig 4A). PDE activity did not change between 6 h and 24 h of exposure time, but ELF-MF exposure significantly attenuated PDE activity compared to that in sham-exposed cells at both 6 h and 24 h of exposure (Fig 4B). These results indicate that the cAMP increase is caused by the attenuation of PDE activity.

### Effect of 24-h ELF-MF exposure on corticosterone secretion depends on the ELF-MF intensity

**Fig 5:**
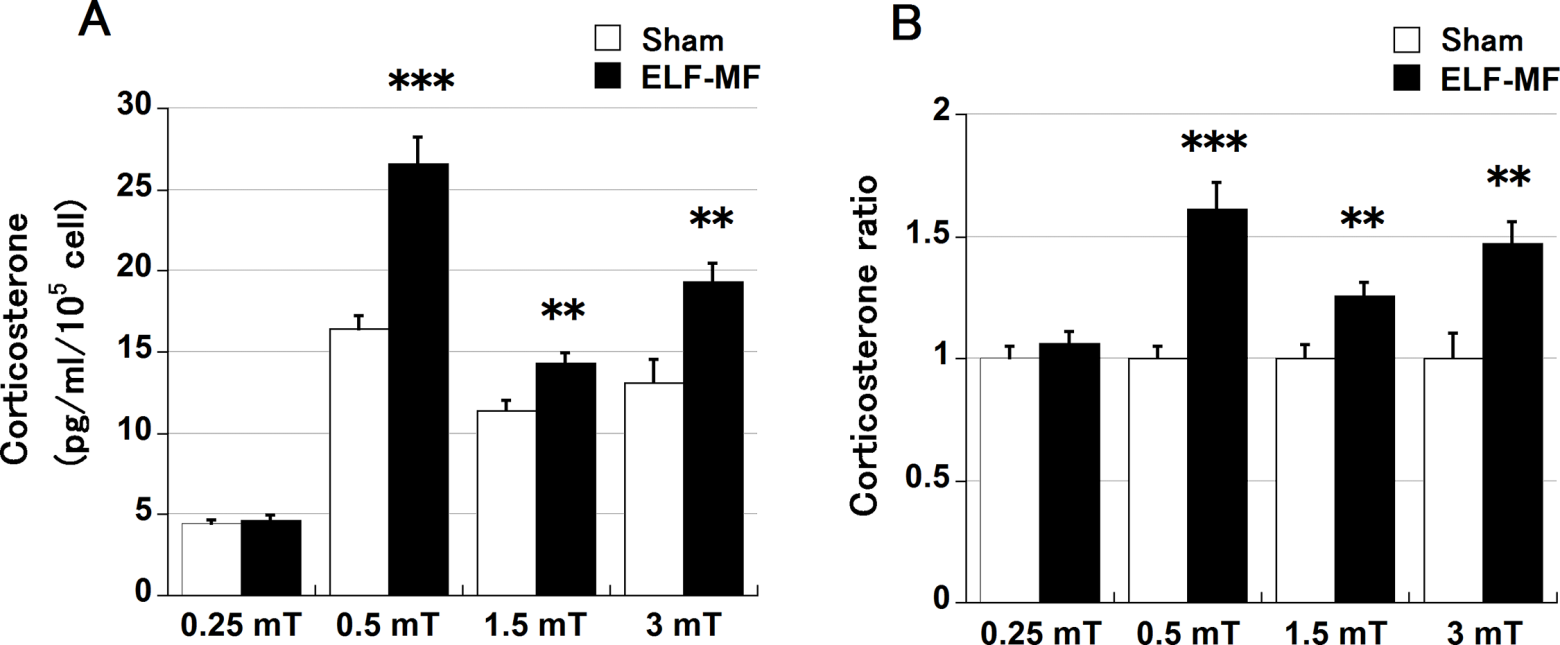
Intensity-dependent effect of ELF-MF exposure on corticosterone secretion. All data are presented as the mean ± S.E.M. Y-1 cells were exposed to ELF-MFs or sham (24-h duration), and the corticosterone concentration in the medium was estimated (n = 8 each). ELF-MF intensities were set 0.25, 0.5, 1.5, and 3 mT, and the corresponding sham exposure in each ELF-MF condition was performed simultaneously. The absolute values (A) and ratios relative to sham exposure (B) are shown. The effect of the ELF-MF was significant at intensities above 0.5 mT. Steroids were quantified twice per sample (dish), **p < 0.01, ***p < 0.001.

We additionally investigated whether the effect of 24-h ELF-MF exposure on corticosterone secretion depended on ELF-MF intensity (Fig 5). The ratios relative to sham exposure (Fig 5B) are also shown because of the change in absolute steroid secretion was significant (p < 0.0001, Fig 5A). The effects of the ELF-MF were significant at intensities above 0.5 mT.

## Discussion

We previously reported increased plasma adrenal steroid levels in mice exposed to an ELF-MF for 200 h, without enhancement of the hypothalamic–pituitary–adrenal axis [9]. Our present results suggested that ELF-MF exposure stimulates adrenal steroidogenesis in the Y-1 cell line, and they strongly support the hypothesis that the ELF-MF stimulates steroidogenesis directly in the adrenal cortex, and not via the common stress response. The significant increase in CYP11A1 (cholesterol side-chain cleavage cytochrome P450), a rate-limiting enzyme for steroidogenesis [13, 14], may have contributed to this increased steroidogenesis.

Human adrenal cortex-derived H295R cells also showed some ELF-MF-triggered changes such as increased mRNA expression of *Star*, a rate-limiting protein for steroidogenesis to facilitate the transport of cholesterol to the inner mitochondrial membrane [12]. However, protein expression of StAR and various other adrenal steroid proteins was not significantly induced by ELF-MF exposure. Non-standardized adrenal steroid concentrations and cell counts for each cell line after both treatments are shown in S1 Fig. The non-standardized steroid secretion data also suggest that the effect in H295R cells is weaker than that in the Y-1 cells (S1B, S1C, S1E and S1F Fig). However, various studies have reported that ELF-MF exposure increases cell proliferation *in vitro* [17–19]. The ELF-MF had a negative, albeit non-significant, effect on the cell count at all time points tested in Y-1 cells, whereas the effect in H295R cells varied for different exposure times (S1A and S1D Fig). The difference in the effects on cell proliferation may have visibly affected the amounts of adrenal steroids because the data of adrenal steroids were presented as concentration/cell count. However, the mechanisms underlying the different proliferative effects in mouse and human cells are unknown at present.

**Fig 6:**
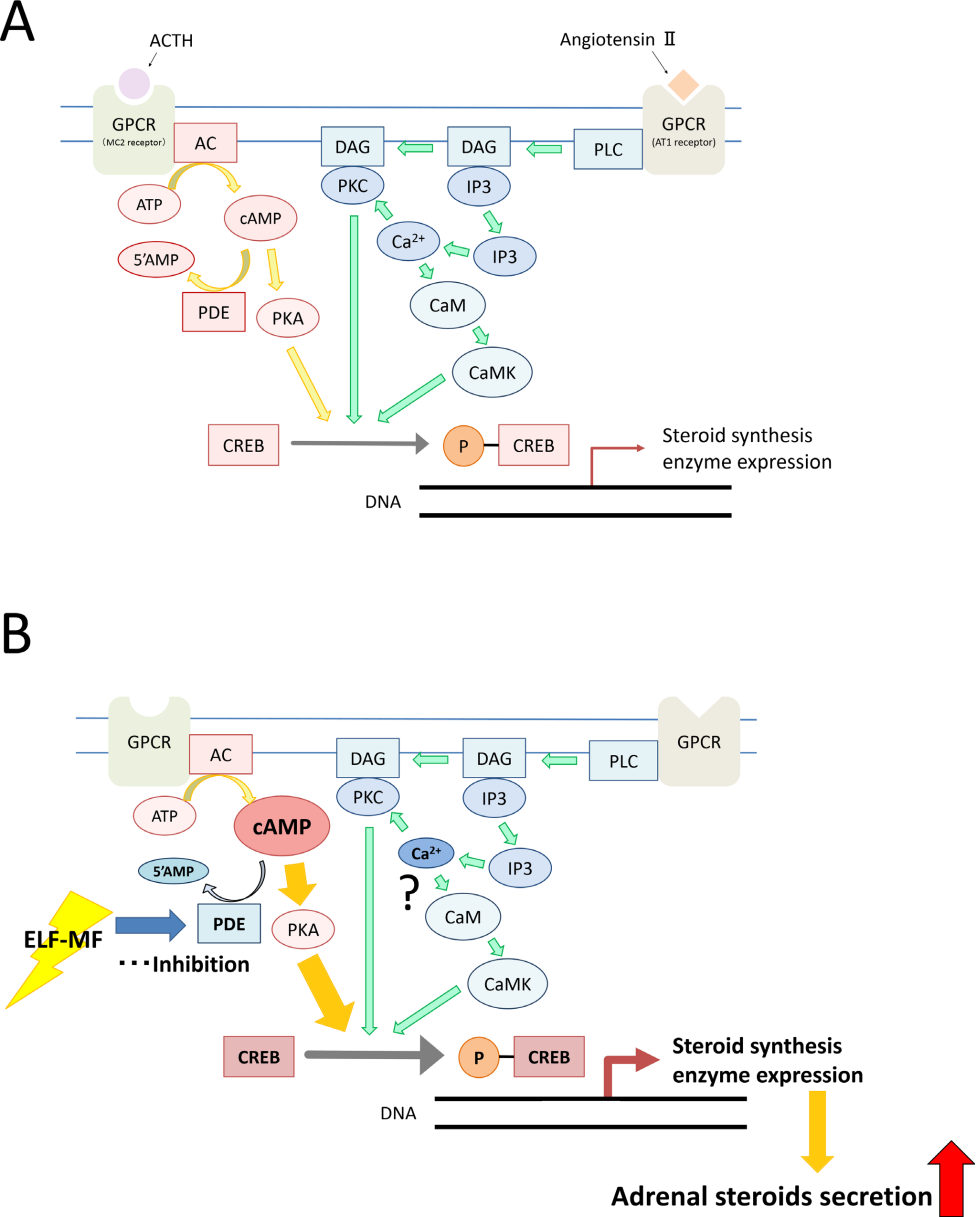
**Signaling pathways regulating adrenal steroidogenesis (A) and the assumed steroidogenic mechanism induced by ELF-MF exposure (B).** AC: adenylate cyclase, cAMP: cyclic adenosine monophosphate, CaM: calmodulin, CaMK: calmodulin kinase, CREB: cAMP response element binding protein, DAG: diacylglycerol, GPCR: G protein-coupled receptor, IP3: inositol triphosphate, PDE: phosphodiesterase, PKA: protein kinase A, PKC: protein kinase C, PLC: phospholipase C, 5′ AMP: adenosine 5'-monophosphate.

ELF-MF-induced adrenal steroidogenesis may activate the cAMP-protein kinase A (PKA)-CREB phosphorylation pathway. This ACTH-stimulated pathway induces gene transcription and synthesis of steroidogenic proteins [13, 14, 20–22]. We investigated the effect of NF449, an inhibitor of the Gsα-subunit, on the increase in cAMP concentration resulting from ELF-MF exposure and PDE activity in cells exposed to the ELF-MF to clarify the mechanism underlying the ELF-MF-dependent increase in cAMP. Treatment with 30 μM NF449 decreased the cAMP concentration in Y-1 cells exposed to sham and ELF-MF treatments; the cAMP-increasing effect of the ELF-MF was preserved. Unexpectedly, the cAMP-increasing effect was weakened by treatment with 10 μM NF449. NF449 is also known as a selective inhibitor of the P2X1 receptor at lower concentrations [23]. Although the P2X1 receptor has not been reported to be expressed in the adrenal cortex [24], an antagonistic effect might affect this result. In contrast, PDE activity significantly decreased in Y-1 cells exposed to the ELF-MF, which suggests that the increase in intracellular cAMP concentration induced by ELF-MF exposure in Y-1 cells resulted from a decrease in PDE activity, and not the activation of G protein-coupled receptors (Fig 6).

Additionally, [Ca^2+^]_i_ was unexpectedly significantly decreased in the cells exposed to the ELF-MF. Although it is well known that increases in [Ca^2+^]_i_ also trigger adrenal steroidogenesis via the angiotensin II-stimulated protein kinase c pathway [16], this effect may be negligible, at least in our study system. The exact mechanism underlying the decrease is unknown, but it may involve feedback regulation in response to the increase in cAMP concentration. In support of this hypothesis, Kaneda et al. [25] reported that PDE inhibitor treatment decreased [Ca^2+^]_i_ in guinea pig ileal longitudinal smooth muscle.

We were not able to determine why ELF-MF exposure inhibited PDE activity in this study. One possible mechanism involves the extracellular-signal-regulated kinase (ERK) pathway. It is known that the PDE4 family, a PDE subgroup, is phosphorylated by ERK2, resulting in a decrease in cAMP-hydrolyzing activity [26]. Moreover, some early studies have reported increased ERK activation upon ELF-MF exposure *in vitro* [14] and in planarian flatworms [27]. PDE inhibition is a powerful pharmacological tool used for treatment of cardiac failure [28] and erectile dysfunction [29]. It is known that the adrenal gland expresses PDE8, contributing to steroidogenesis [20–22]. If the PDE-inhibitory effect has subtype specificity, ELF-MF exposure may possess potential for hypoadrenalism therapy. Further investigation is needed to clarify the PDE inhibition mechanism and subtype specificity.

We additionally investigated whether the effect of ELF-MF exposure on corticosterone secretion is intensity-dependent. The effect of the ELF-MF was significant at intensities above 0.5 mT. This intensity is above the reference level of 0.2 mT for general public exposure to a 60-Hz ELF-MF as proposed by the International Commission on Non-Ionizing Radiation Protection; however, it is below the reference level for occupational exposure, which is set at 1 mT [30]. Our results obtained from the human-derived H295R cells suggest that the resistance in humans against ELF-MF exposure is higher than that in mice. However, the possibility of enhancement of adrenal steroid secretion by ELF-MF exposure in the case of occupational exposure should be investigated in more detail.

In conclusion, ELF-MF exposure stimulates adrenal steroidogenesis via an increase in intracellular cAMP, which is caused by inhibition of PDE activity in mouse adrenal cortex-derived Y-1 cells. The same mechanism may trigger the increase in adrenal steroid secretion in mice observed in our previous study [9]. The impact of our findings on human health is currently unclear because the data in this study were obtained from human-derived cell cultures. More research regarding the influence of occupational exposure and the therapeutic potential of the ELF-MF is required in the future.

## Supporting Information

S1 Fig Non-standardized adrenal steroid concentrations and cell counts in ELF-MF and sham-exposed Y-1 and H295R cells. All data are presented as the mean ± S.E.M. (A) The ELF-MF effects for cell counts were negative at all-time points in Y-1 cells, although not significantly. (D) In contrast, H295R cells showed some variation among exposure times. In the 6-h exposure, the cell count was lower in the ELF-MF group than in the sham group. Corticosterone (B) and aldosterone (C) levels of Y-1 cells were significantly higher after 6 h of ELF-MF exposure than in the sham group. However in H295R, no significant effect of ELF-MF was observed for cortisol (E), and a significant effect on aldosterone secretion was noted only after 24 h of ELF-MF exposure (F). Cell counts and sampling of medium were performed for each sample (culture dish). Steroids were quantified twice per sample. n = 8 each, *p < 0.05, **p < 0.01, ***p <0.001.
(DOCX)
