# Supplementary material for: Exposure to an Extremely-Low-Frequency Magnetic Field Stimulates Adrenal Steroidogenesis via Inhibition of Phosphodiesterase Activity in a Mouse Adrenal Cell Line
Source: PLoS One. 2016 Apr 21;11(4):e0154167. doi: 10.1371/journal.pone.0154167 (PMC4839720; doi:10.1371/journal.pone.0154167)
Supplement: S1 Fig — All data are presented as the mean ± S.E.M. (A) The ELF-MF effects for cell counts were negative at all-time points in Y-1 cells, although not significantly. (D) In contrast, H295R cells showed some variation among exposure times. In the 6-h exposure, the cell count was lower in the ELF-MF group than in the sham group. Corticosterone (B) and aldosterone (C) levels of Y-1 cells were significantly higher after 6 h of ELF-MF exposure than in the sham group. However in H295R, no significant effect of ELF-MF was observed for cortisol (E), and a significant effect on aldosterone secretion was noted only after 24 h of ELF-MF exposure (F). Cell counts and sampling of medium were performed for each sample (culture dish). Steroids were quantified twice per sample. n = 8 each, *p < 0.05, **p < 0.01, ***p <0.001. (DOCX) [file pone.0154167.s001.docx]

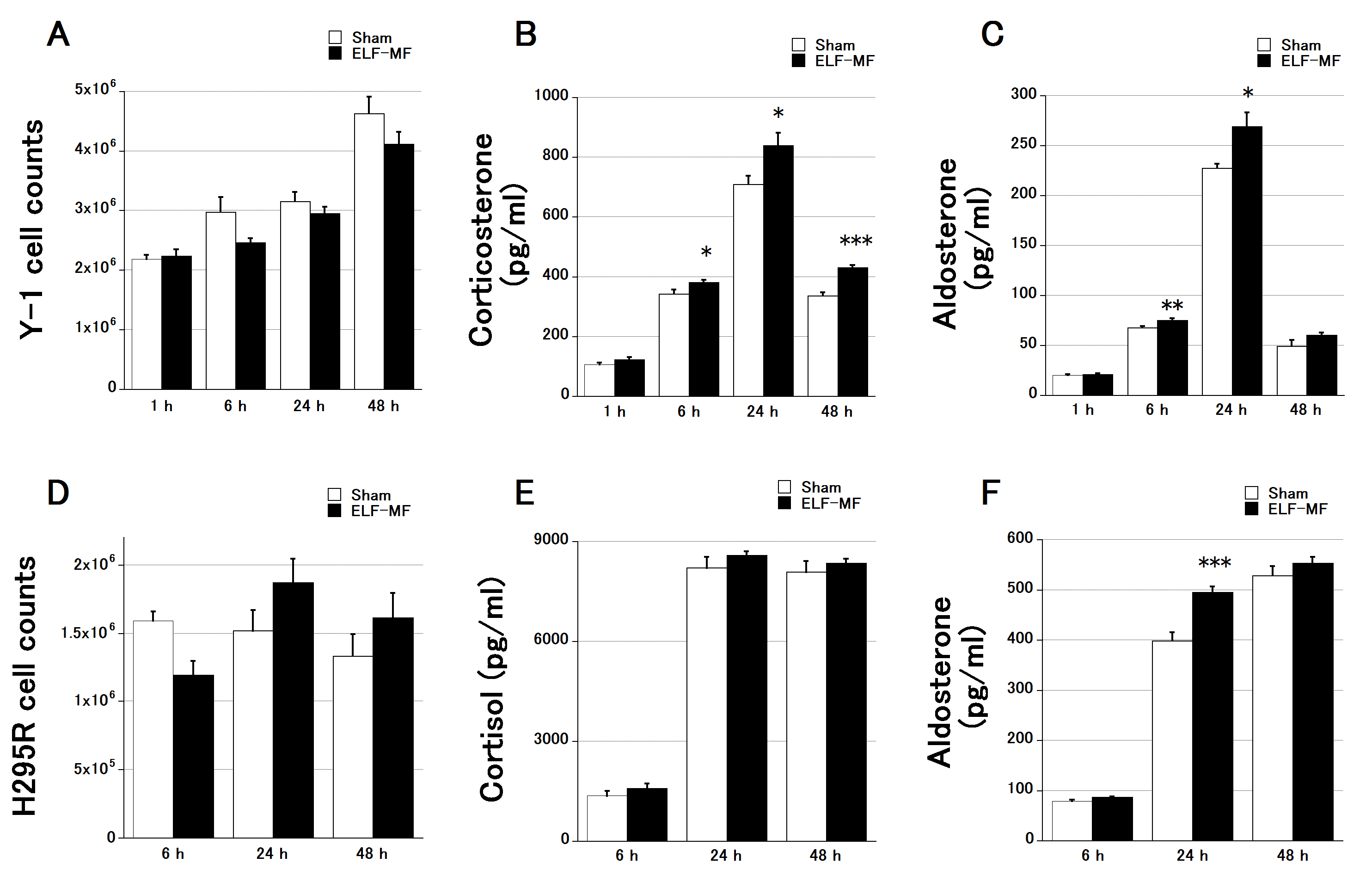


**Fig. S1** **Non-standardized adrenal steroid concentrations and cell counts in ELF-MF- and sham-exposed Y-1 and H295R cells.** All data are presented as the mean ± S.E.M. (A) The ELF-MF effects for cell counts were negative at all-time points in Y-1 cells, although not significantly. (D) In contrast, H295R cells showed some variation among exposure times. In the 6-h exposure, the cell count was lower in the ELF-MF group than in the sham group. Corticosterone (B) and aldosterone (C) levels of Y-1 cells were significantly higher after 6 h of ELF-MF exposure than in the sham group. However in H295R, no significant effect of ELF-MF was observed for cortisol (E), and a significant effect on aldosterone secretion was noted only after 24 h of ELF-MF exposure (F). Cell counts and sampling of medium were performed for each sample (culture dish). Steroids were quantified twice per sample. n = 8 each, *p < 0.05, **p < 0.01, ***p <0.001.
